# Epidemiology and Viral Etiology of Pediatric Immune Thrombocytopenia through Korean Public Health Data Analysis

**DOI:** 10.3390/jcm10071356

**Published:** 2021-03-25

**Authors:** Jae Hee Lim, Yu Kyeong Kim, So Hyeon Min, Sang Won Kim, Young Hwan Lee, Jae Min Lee

**Affiliations:** 1 Department of Medicine, College of Medicine, Yeungnam University, Daegu 42415, Korea; imjh520@naver.com (J.H.L.); dbrud21206@naver.com (Y.K.K.); 3000min@naver.com (S.H.M.); 2 Medical Research Center, College of Medicine, Yeungnam University, Daegu 42415, Korea; kimsw3767@ynu.ac.kr; 3 Department of Pediatrics, College of Medicine, Yeungnam University, Daegu 42415, Korea

**Keywords:** immune thrombocytopenia, viral infection, children

## Abstract

Immune thrombocytopenic purpura (ITP) is prevalent in children aged 2–5 years but may occur in all pediatric age groups. In 50–60% of pediatric patients, ITP is preceded by an upper respiratory tract infection 1–4 weeks before its onset. In this study, the relationship between the development of ITP and viral infections in children was assessed. We analyzed data of 6487 patients aged < 18 years with incident ITP from the Health Insurance Review and Assessment Open Access Big Data Platform (2015 to 2018) and the Korea Disease Control and Prevention Agency. The monthly positive detection rate (PDR) of seven respiratory and four acute diarrhea viruses was calculated. The virus PDR seasonal trend data was analyzed through ARIMA modeling. The ITP diagnostic data and prevalence of viral infection 1 and 2 months prior were analyzed using the Granger test. The overall male to female (M/F) ratio was 1.2, whereas it was 1.4 in the youngest age group (< 1 year). The overall ITP incidence rate was 18.1 per 100,000 person-years. Respiratory syncytial virus, rhinovirus, rotavirus, and astrovirus infections influenced ITP occurrence in children. However, rotavirus infection is positively associated with the etiology of ITP after 1–2 months.

## 1. Introduction

Immune thrombocytopenic purpura (ITP) is a disorder in which the primary hemostasis process is impaired due to low platelet numbers. The incidence rate of ITP in children is known to be 1.6–5.3 per 100,000 person-years [1,2,3,4,5,6,7,8]. In a recent Korean study, the ITP incidence rate for all ages was 5.3 per 100,000 person-years, while it was 14.3 per 100,000 person-years for children aged < 15 years. Although prevalent in children aged < 1 year, it can occur at any pre-adolescent age [9].

Unlike the common form of ITP found in adults, ITP in children generally recovered with or without treatment within 6 to 12 months after diagnosis. However, 20–25% of pediatric patients suffer from chronic ITP [10,11].

It is well-known that ITP pathogenesis involves infection. Viruses such as Epstein-Barr virus (EBV), hepatitis C virus (HCV), and human immunodeficiency virus (HIV) have been associated with ITP [12,13,14,15]. In some cases, live vaccines such as measles, mumps, rubella, or administration of drugs like acetazolamide and aspirin, also cause infections [16]. An upper respiratory infection precedes ITP by 1–4 weeks in 50–65% of pediatric patients.

The cause of ITP is known to be associated with infection, but there are no studies on its association with respiratory or gastrointestinal viruses. The purpose of our study is to find a clue about the exact etiology of ITP through the virus associated with ITP incidence, and to help to understand the pathogenesis of ITP. Barring a few case reports, the association between respiratory viruses and pediatric ITP is not well-studied. We aimed to analyze public health data provided by the Health Insurance Review and Assessment (HIRA) Open Access Big Data Platform and Korea Disease Control and Prevention Agency (KDCA) to determine the relationship between respiratory and gastroenteritis viruses and ITP.

## 2. Materials and Methods

### 2.1. HIRA Databases

The National Health Insurance (NHI) system is the only public medical insurance system operated by the Ministry for Health, Welfare, and Family Affairs in Korea. This universal health coverage system covers approximately 98% of the overall Korean population. The HIRA is a government-affiliated organization created to build an accurate claims review and quality assessment system for the NHI. HIRA databases are open for all investigators with academic purposes. Claims data in the HIRA database include patient diagnosis, treatment, procedures, surgical history, and prescription drugs, serving as a valuable resource for healthcare service research [17,18,19].

### 2.2. Study Population

**Figure 1:**
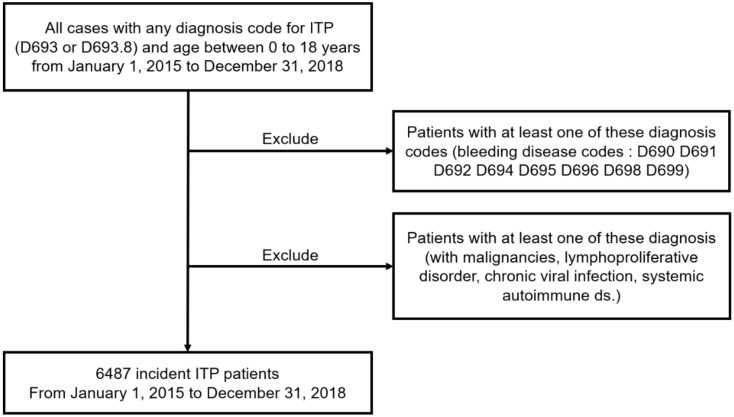
Flowchart illustrating patient selection. ITP, immune thrombocytopenia.

The study population included ITP patients aged <18 years. Diagnoses were coded according to the 7th Korean Classification of Disease (KCD-7), a modified version of the International Classification of Disease-10 (ICD-10). The selection process followed several steps (Figure 1). First, data of patients diagnosed with ITP (KCD-7 Codes D693 or D693.8) as the primary or related disease, from 2015 to 2018, were extracted from HIRA. Next, cases that shared other bleeding disease codes, starting with D69 after the first ITP code, were dropped. These codes were D690, D691, D692, D694, D695, D696, D698, or D699 (Supplemental Table S1). Patients with ITP-associated malignancies, including lymphoproliferative disorders, chronic viral infection, or systemic autoimmune diseases, were classified as secondary ITP, according to an international working group, and were excluded from the study (Supplemental Table S2) [20]. Therefore, a total of 6487 incident ITP cases between 1 January 2015, and 31 December 2018, were included in this study.

### 2.3. The Surveillance Data of Virus 

We used data on viruses that cause acute respiratory infection and gastroenteritis, reported by the KDCA. More than 4000 respiratory and 2000 enteric specimens were collected from 17 local environmental and health institutes and over 100 participating hospitals across Korea during each year of the study period. The causative pathogens were identified using standardized diagnostic procedures in a central laboratory. Pathogen prevalence was surveyed weekly and analyzed based on genetic testing of patients with influenza-like illness or acute diarrhea. We collected the positive detection rate (PDR) data from 2015 to 2018 and calculated the average monthly PDRs of seven respiratory viruses (adenovirus (HAdV), parainfluenza virus (HPIV), respiratory syncytial virus (HRSV), influenza virus (IFV), coronavirus (HCoV), rhinovirus (HRV), and bocavirus (HBoV)) and four acute diarrhea viruses (adenovirus, rotavirus, norovirus, and astrovirus).

### 2.4. Statistical Analyses 

We constructed a model of seasonal variations in ITP diagnosis using the autoregressive integrated moving average (ARIMA) modeling approach. The ARIMA model assumes that the current observation is related to past observations through time. The general multiplicative form of the ARIMA model is denoted as (p, d, q), where p, d, q are the order values of non-seasonal autoregressive, differencing, and moving average parameters, respectively. The Autocorrelation Function (ACF) was examined to identify the general form of the model to be fit. Considering the ACF graphs, different ARIMA models were identified for model selection (Supplemental Figures S1 and S2). The minimum Akaike’s information criterion (AIC) model was chosen as the best-fit model (Supplemental Table S4). The Granger approach was used to investigate how many of the current values in the time series y could be described as other values in the time series y [21]. The data were analyzed using the R software. Seasonal differences in newly diagnosed childhood ITP were analyzed using the chi-square test, and *p*-values < 0.05 were considered statistically significant.

## 3. Results

### 3.1. Patient Characteristics

During the 4-year-period from 2015–2018, 6487 patients aged < 18 years were diagnosed with ITP (Table 1). Of these, 2470 (38.1%) patients were aged < 1 year, 1895 (29.2%) were aged 1.1–5 years, and 2122 (32.7%) were aged 5.1–18 years. The overall male to female (M/F) ratio was 1.2, whereas the M/F ratio was 1.4 in the youngest age group (<1 year) and 1.1 in 1–5 and 5–18 years age groups. The overall ITP incidence rate was 18.1 per 100,000 person-years. According to age stratification, ITP incidence rates were 145.4, 25.7, and 7.9 person-years in the 0–1, 1–5, and 5–18 age groups.

### 3.2. Trend Analysis of ITP

**Figure 2:**
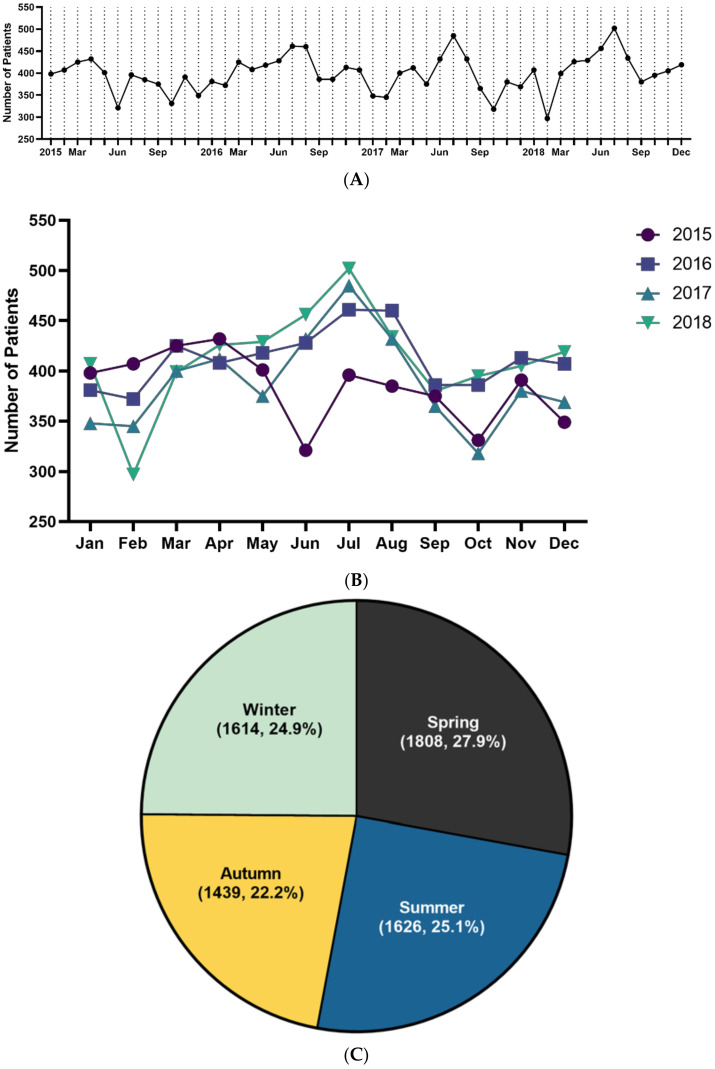
Trend of ITP incidence (**A**). Monthly trend analysis of immune thrombocytopenia from 2015 to 2018 (**B**). Monthly trend analysis of immune thrombocytopenia according to year (**C**). Seasonal trend of immune thrombocytopenia incidence. Spring (March to May), summer (June to August), autumn (September to November), and winter (December to February) (cumulative incidence for 4 years, %).

Of the total 6487 patients, 1659 were diagnosed in 2015, 1705 in 2016, 1538 in 2017, and 1585 in 2018. The highest incidence rate in 2015 and 2017 was in April, and in 2016 and 2018, it was highest in June (Supplementary Table S3). The annual cumulative cases per month were the highest in April and lowest in October (Figure 2A,B). ITP patients were most often diagnosed during spring (27.9%), followed by summer (25.1%), winter (24.9%), and autumn (22.2%) (Figure 2C). The average number of cases per month was 135.1. The average number of cases per year between 2015 and 2018 was 1621.8.

### 3.3. Positive Detection Rates of Virus

The PDRs of most viruses showed seasonal variation (Supplemental Table S5). HAdV was highest from September to November, HPIV was highest in May, HRSV was highest from November to December, IFV was highest from January to February, and HCoV and norovirus were highest from December to January, while HRV and enteric HAdV were highest in September, bocavirus, HMPV, and rotavirus were highest in May, April, and March, respectively. PDR of astrovirus was highest in January. PDR of all viruses was highest in December and lowest in July.

### 3.4. Causality between ITP and Virus Prevalence

If any virus prevalent time affected ITP diagnosis, the prevalence of that virus might increase before a peak of ITP diagnosis. Thus, a Granger causality test was conducted between the virus PDR data and the ITP diagnostic data from 1 to 2 months later. The results of this Granger causality test are shown in Table 2 and Table 3. Among the seven respiratory viruses and four gastrointestinal viruses analyzed, the prevalence of some viruses increased 1 to 2 months before ITP incidence increases. The PDR for rotavirus (*p* = 0.035) in < 5-year-old patients correlated with increased ITP incidence after 1 month (Table 2). Moreover, PDRs for HRSV (*p* = 0.030) and astrovirus (*p* = 0.029) in the patients aged 5.1–18 years correlated with increased ITP prevalence after 1 month. The PDR for HRV (*p* = 0.015) and rotavirus (*p* = 0.050) in patients aged 0–1 years, and rotavirus (*p* = 0.031) in patients < 5 years of age co-related with increased ITP prevalence after 2 months (Table 3).

## 4. Discussion

This study evaluated ITP incidence and its association with viral infections. We found that some respiratory and gastroenteritis viruses positively affected ITP incidence after 1 and 2 months, respectively.

A French nationwide study evaluated 3771 ITP patients of all age groups for 2 years (2009–2011). The incidence rate was 2.83 per 100,000 person-years in patients aged < 18 years, with peaks among children aged < 5 years and the elderly aged > 60 years [22]. A recent Japanese study included 7774 ITP cases from 2004 to 2007 and reported a marked predominance of boys among newborns and infants in the < 4 years age group [23]. In that study, the M/F ratio in total patients was 0.64, and those in children aged <4 years was 1.45. The incidence rate was 1.91 per 100,000 person-years in the < 14 years age group. In a previous Korean study that analyzed the HIRA database, 10,814 ITP patients were diagnosed, and the incidence rate was 5.3 per 100,000 person-years for 4 years (2010–2014) [9]. There were 3798 ITP cases in the < 15 years age group with an incidence rate of 14.3 person-years; ITP incidence was more common in males than females in the < 10 years age group. In our study, ITP incidence by age and sex were similar to the previous Korean study. 

Recently, autoimmune disorders were reported to be the most common cause of acute childhood ITP. Autoantibodies on the platelet surface are known to be generated by viral antigens [11,24]. Viruses, including CMV, HCV, EBV, varicella virus, herpes virus, HIV, and IFV, are associated with the development of ITP [12,14,25,26,27]. In our study, we found that, among the respiratory viruses, infection with HRSV or HRV was associated with the development of ITP.

Except for a few case reports, few studies analyze the association between ITP and respiratory viral infections. Pokhrel et al. reported the case of a 28-year-old male who developed RSV infection 19 months after double umbilical cord transplantation for acute lymphoblastic leukemia. The patient was treated with aerosolized ribavirin, and at the same time, thrombocytopenia was detected [28].

Meanwhile, HRV infection has been reported to be associated with ITP in two cases. Both cases were reported in the US; in a 10-year-old child and a 29-year-old young adult. The young adult patient was diagnosed with ITP due to HRV infection [29], and the child was co-infected with SARS-CoV-2 and HRV/Enterovirus at ITP diagnosis [30].

IFV is one of the viruses known to be associated with ITP. There are several case reports on a correlation between IFV A infection and ITP [31,32], and although rare, an IFV B infection has also been reported to be related to ITP [33]. However, in our study, there was no statistical significance between the PDR of IFV and ITP incidence rates (1-month interval, *p* = 0.496; 2-month interval, *p* = 0.466).

In our opinion, although the number of patients infected with IFV increase rapidly during IFV outbreaks, their platelet count is likely to be underdiagnosed because many subclinical and non-severe ITPs are not diagnosed during hospital visits.

Moreover, there have been cases demonstrating a correlation between coronavirus and Zika virus infections and ITP [34,35]. Some studies have reported the association between respiratory viral infection and the development of ITP; however, studies on the association between viral gastroenteritis and ITP are few. In our study, ITP showed the highest prevalence during spring. Besides, 1 and 2 months after detection, rotavirus and astrovirus showed causality in ITP incidence.

A Chinese group reported that rotavirus infections were responsible for the occurrence of ITP [36]. In China, rotavirus infections were common in winter [37]. ITP-affected children with rotavirus infection were significantly younger than those without rotavirus infection, and most cases of ITP with rotavirus infection occurred during winter, whereas ITP cases without rotavirus infection showed no seasonal variation. In Korea, rotavirus infection was significantly reduced due to the introduction of rotavirus vaccines in the 2000s [38]. Therefore, the difference in the ITP seasonal occurrence trend between Korea and China is considered to be due to the higher prevalence of rotavirus infections in China.

Rotavirus antibodies are detected in blood serum within 2 days after the onset of diarrhea [38]. The action of rotavirus antibodies on platelets is presumed to be the mechanism by which rotavirus-related ITP is induced. Furthermore, astrovirus showed meaningful results in our study; however, there have been no previous studies on the association between astrovirus infections and ITP incidence. During astrovirus infection, antibodies targeting the astrovirus capsid protein are produced in the host [39,40]. Therefore, astrovirus could also cause ITP, even though no cases have been reported so far.

In previous studies, there has been a focus on the association between ITP and viruses such as CMV, HIV, and HCV, which are important viruses [15], but no studies have studied the association between respiratory or gastroenteritis viruses and ITP. The reason for this seems to be the lack of technology for extraction and identification of respiratory and gastroenteritis viruses. Blood tests for CMV, HIV, and HCV can be used to detect viral infections, but respiratory viral infections or viral gastroenteritis cannot be confirmed by blood tests alone. With the recent development of multiplex PCR (mPCR), testing for many infectious diseases including respiratory infections, infectious gastroenteritis, and central nervous system infections has become simple. Rapid tests are possible because many pathogens can be detected through a single test [41,42]. Therefore, we tried to study the correlation with ITP using respiratory viruses and gastrointestinal viruses that can be quickly tested through mPCR. 

There are some limitations to this study. First, it is a retrospective study. Therefore selection bias may occur and may have an inferior level of evidence compared with prospective studies. Second, there is a different between the patients tested for viral infections and patients diagnosed with ITP. Thus, a direct relationship could not be established between viral infection and ITP because only the predecessor relationship could be assessed through the incidence rate. However, the diagnostic trend of ITP and the virus detection rate were shown in a time series graph, and the relationship between them was analyzed through the Granger causality test.

## 5. Conclusions

This study evaluated ITP incidence and investigated the correlation between ITP diagnosis and the prevalence of common viral respiratory infections and gastroenteritis. Although there have been several previous studies, most were performed among a small number of patients at single institutions or involved limited periods. To our knowledge, this is the largest nationwide analysis of pediatric ITP patients and their association with the PDRs of common viruses in Korea. Our findings suggest that HRSV, HRV, rotavirus, and astrovirus infections influence the pathogenesis of ITP in children. These results show that clinicians will be able to provide early diagnosis and appropriate treatment of ITP to patients infected with HRSV, HRV, rotavirus, and astrovirus through careful follow-up. Prospective studies on these respiratory and gastrointestinal viruses and ITP will be needed in the future.

## Figures and Tables

**Table 1:** Characteristics of patients.

Age Group, *n* (%)	0–1 Year	1.1–5 Years	5.1–18 Years	Total
Total	2470 (38.1)	1895 (29.2)	2122 (32.7)	6487 (100.0)
Male	1433 (22.1)	1010 (15.6)	1087 (16.8)	3530 (54.4)
Female	1037 (16.0)	885 (13.6)	1035 (15.9)	2957 (45.6)
Male to female ratio	1.4	1.1	1.1	1.2
Incidence rate	145.4	25.7	7.9	18.1

**Table 2:** Causality of immune thrombocytopenia (ITP) incidence after 1 month with virus.

	0–1 Year	1.1–5 Year	<5 Year	5.1–18 Year
HAdV	0.532	0.881	0.831	0.353
HPIV	0.619	0.560	0.992	0.869
HRSV	0.696	0.918	0.772	0.030
IFV	0.599	0.924	0.782	0.996
HCoV	0.522	0.764	0.564	0.065
HRV	0.052	0.469	0.475	0.312
HBoV	0.493	0.489	0.960	0.887
HMPV	0.082	0.891	0.396	0.202
Rotavirus	0.208	0.085	0.035	0.837
Norovirus	0.832	0.571	0.873	0.079
Adenovirus	0.385	0.486	0.319	0.482
Astrovirus	0.646	0.871	0.169	0.029

HAdV, human adenovirus; HPIV, human parainfluenza virus; HRSV, human respiratory syncytial virus; IFV, influenza virus; HCoV, human coronavirus; HRV, human rhinovirus; HBoV, human bocavirus; HMPV, human metapneumovirus.

**Table 3:** Causality of Immune Thrombocytopenia (ITP) incidence after 2 months with virus.

	0–1 Year	1.1–5 Year	<5 Year	5.1–18 Year
HAdV	0.638	0.893	0.947	0.411
HPIV	0.885	0.840	0.836	0.997
HRSV	0.885	0.426	0.517	0.063
IFV	0.472	0.350	0.871	0.242
HCoV	0.133	0.766	0.251	0.241
HRV	0.015	0.708	0.274	0.370
HBoV	0.706	0.369	0.513	0.995
HMPV	0.114	0.176	0.090	0.487
Rotavirus	0.050	0.093	0.031	0.877
Norovirus	0.065	0.972	0.375	0.314
Adenovirus	0.518	0.266	0.503	0.212
Astrovirus	0.104	0.761	0.493	0.015

## Data Availability

The data presented in this study are available on request from the corresponding author.

## Supplementary Materials

The following are available online at https://www.mdpi.com/2077-0383/10/7/1356/s1, Figure S1: Residual ACF correlogram and 95% confidence limits for newly diagnosed pediatric immune thrombocytopenia, Figure S2: Residual ACF correlogram and 95% confidence limits according to the virus, Figure S3. Correlation between incidence of immune thrombocytopenia and virus positive detection rate. Table S1: Diseases and codes of purpura and other hemorrhagic conditions, Table S2: Causes of secondary immune thrombocytopenia, Table S3: Monthly numbers of newly diagnosed pediatric immune thrombocytopenia (ITP) patients in Korea, Table S4: Parameters of ARIMA models for ITP patients by age, Table S5: Positive detection rates of viruses during the study period.
