# Supplementary material for: Epidemiology and Viral Etiology of Pediatric Immune Thrombocytopenia through Korean Public Health Data Analysis
Source: J Clin Med. 2021 Mar 25;10(7):1356. doi: 10.3390/jcm10071356 (PMC8037772; doi:10.3390/jcm10071356)
Supplement: Supplementary file 1 [file jcm-10-01356-s001.pdf]

Supplementary Data

Figure S1. Residual ACF (Auto Correlation Function) correlogram and 95% confidence limits for newly diagnosed pediatric immune thrombocytopenia.

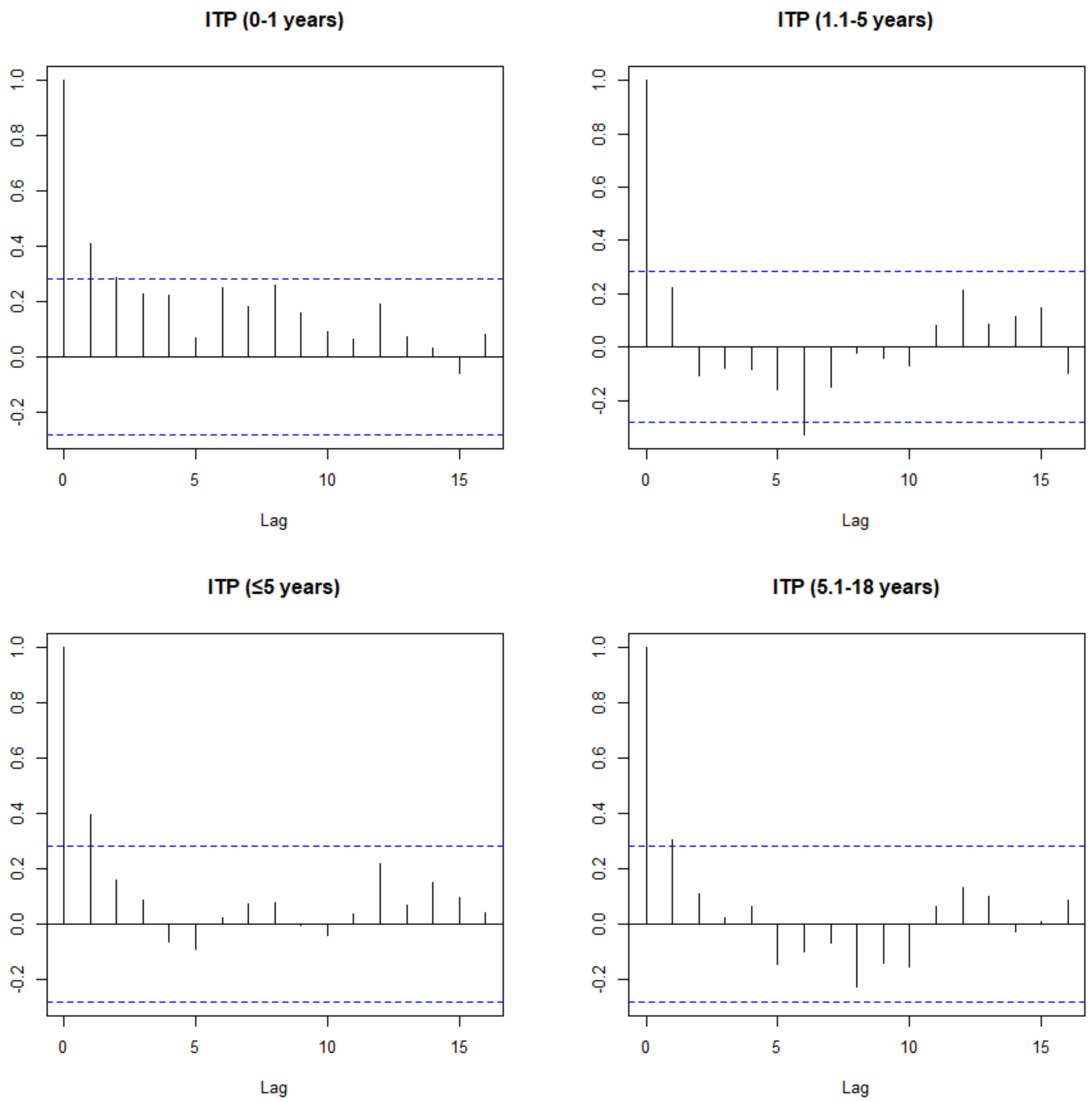

ITP, immune thrombocytopenia

Figure S2. Residual ACF correlogram and 95% confidence limits according to virus.

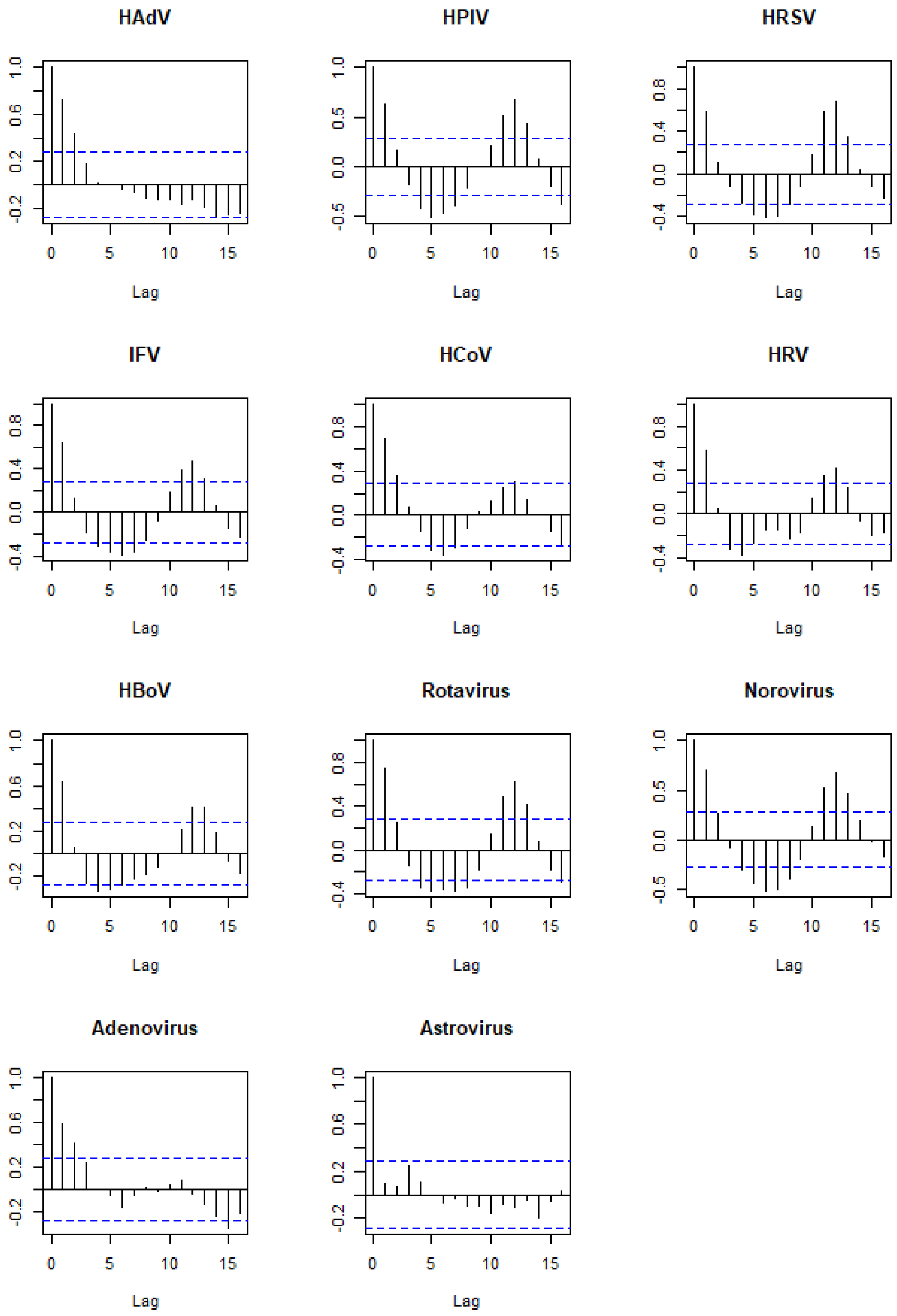

HAdV, adenovirus; HPIV, parainfluenza virus; HRSV, human respiratory syncytial virus; IFV, influenza virus; HCoV, coronavirus; HRV, rhinovirus; HBoV, bocavirus

Figure S3. Correlation between incidence of immune thrombocytopenia and virus positive detection rate

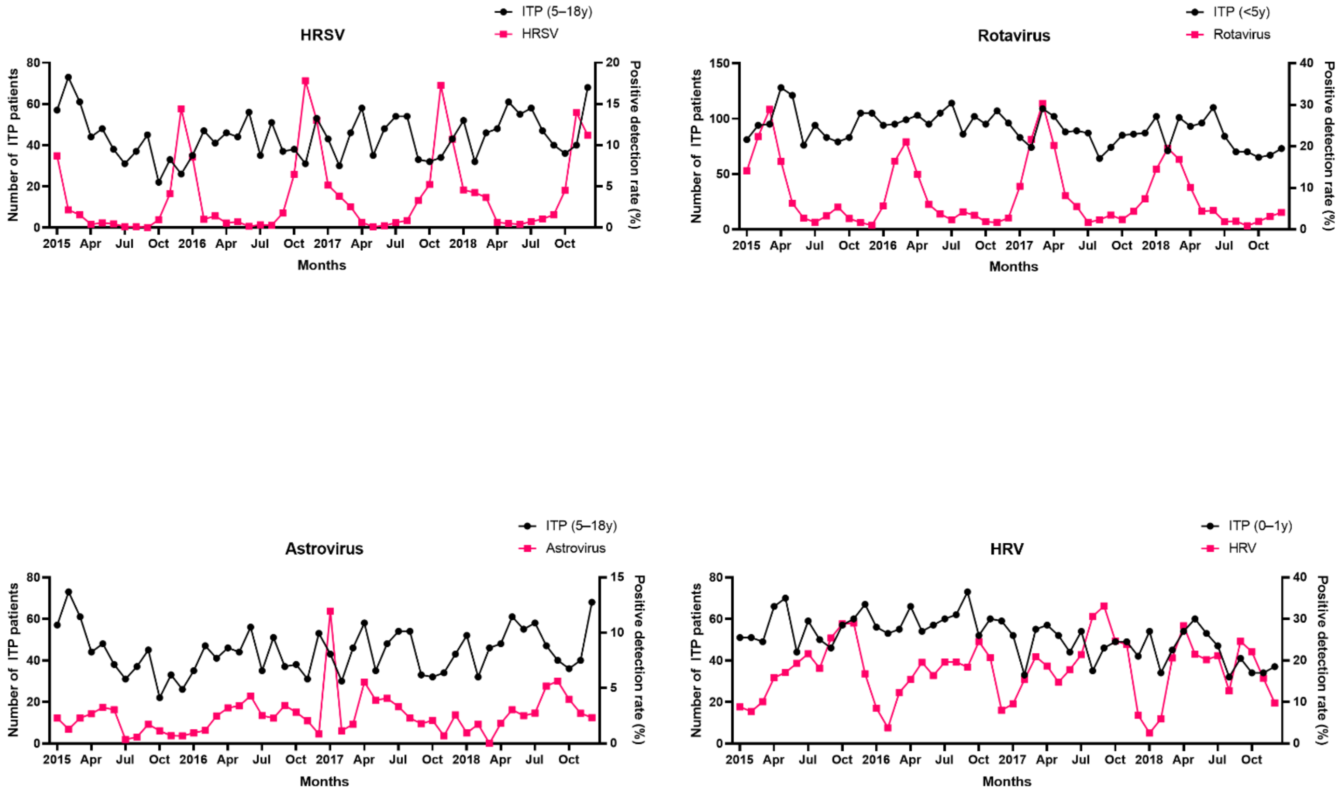

ITP, immune thrombocytopenia; HRSV, human respiratory syncytial virus; HRV, rhinovirus

Table S1. Diseases and codes of purpura and other hemorrhagic conditions.

Diagnosis	Code
Allergic purpura	D690
Anaphylactoid purpura	
Henoch-Schönlein purpura	
Haemorrhagic nonthrombocytopenic purpura	
Idiopathic nonthrombocytopenic purpura	
Vascular purpura	
Vasculitis, allergic	
Qualitative platelet defects	D691
Bernard-Soulier (giant platelet) syndrome	
Glanzmann's disease	
Grey platelet syndrome	
Thromboasthenia (haemorrhagic, hereditary)	
Thrombocytopathy	
Qualitative platelet defects	D692
Bernard-Soulier [giant platelet] syndrome	
Glanzmann's disease	
Grey platelet syndrome	
Evans syndrome	D6930
Other primary thrombocytopenia	D694
Secondary thrombocytopenia	D695
Thrombocytopenia, unspecified	D696
Other specified hemorrhagic conditions	D698
Capillary fragility (hereditary)	
Vascular pseudohaemophilia	
Hemorrhagic condition, unspecified	D699

Table S2. Causes of secondary immune thrombocytopenia.

Diagnosis	Code
Malignancies	
In situ neoplasms	D00–D09
Malignant neoplasms	C00–C97
Hematological malignancies	C77, C81–C96
Lymphoma	C915, C81–C88
Hodgkin lymphoma	C81
B-cell chronic lymphocytic leukemia	C911
Multiple myeloma and plasma cell neoplasms	C90
Waldenström macroglobulinaemia	C880
Myelodysplastic syndromes	D46
Infection or immunodeficiency	
Viral hepatitis C or B	B16, B171, B180–B182
Viral hepatitis C	B171, B182
Viral hepatitis B	B16, B180-B181
Human immunodeficiency virus disease	B20–B24
Immunodeficiency	D80–D84
Autoimmune disease	
Connective tissue disease	M32-M351
Systemic lupus erythematosus	M32
Systemic sclerosis	M34
Dermatopolymyositis	M33
Sicca syndrome	M350
Mixed connective tissue disease	M351
Rheumatoid arthritis	M05, M060, M062–M063, M068–M069, M080
Other autoimmune hemolytic anemias	D591
Antiphospholipid syndrome	D686
Sarcoidosis	D86

Table S3. Monthly numbers of newly diagnosed pediatric immune thrombocytopenia (ITP) patients in Korea.

	Jan	Feb	Mar	Apr	May	Jun	Jul	Aug	Sep	Oct	Nov	Dec	Total	Aver-age
2015	138	167	156	172	169	114	125	120	124	105	138	131	1659	138.3
2016	129	142	140	149	139	161	149	137	139	133	138	149	1705	142.1
2017	126	104	155	160	123	137	141	118	107	117	120	130	1538	128.2
2018	154	103	147	141	157	165	142	117	110	101	107	141	1585	132.1
Total	547	516	598	622	588	577	557	492	480	456	503	551	6487	540.6
Average	136.8	129.0	149.5	155.5	147.0	144.3	139.3	123.0	120.0	114.0	125.8	137.8	1621.8	135.1

Table S4. Parameters of ARIMA models for ITP patients by age.

Parameters.	0–1 Years	1.1–5 Years	5.1–18 Years	Total
p autoregressive	1	1	1	1
d difference	0	0	0	0
q moving average	1	2	4	0
AIC	342.81	344.95	366.96	490.1

AIC, Akaike's Information Criterion.

Table S5. Positive detection rates of viruses during the study period.

Mon / PDR (%)	Jan	Feb	Mar	Apr	May	Jun	Jul	Aug	Sep	Oct	Nov	Dec
**HAdV**												
2015	5.04	2.15	2.83	4.70	4.83	5.63	3.32	3.18	2.30	6.98	9.03	8.78
2016	9.18	3.88	3.93	5.54	6.53	6.95	7.16	9.48	9.58	6.50	5.83	3.24
2017	3.72	2.53	2.43	2.74	6.05	4.73	2.96	5.18	4.33	3.78	4.73	2.60
2018	2.98	2.80	4.25	5.38	5.45	6.80	5.82	9.55	13.33	11.30	10.70	7.63
**HPIV**												
2015	0.92	0.55	0.78	6.30	17.65	11.75	7.04	6.28	8.93	4.72	9.53	4.08
2016	2.24	1.30	1.40	5.60	16.55	16.23	9.68	10.25	8.68	4.56	1.95	0.78
2017	1.58	1.70	2.98	8.38	19.43	12.83	9.68	8.95	4.60	3.04	3.33	2.68
2018	0.84	1.60	3.25	9.44	19.10	16.78	11.12	7.75	4.33	4.00	1.75	1.03
**HRSV**												
2015	8.70	2.15	1.55	0.40	0.58	0.45	0.12	0.13	0.00	0.96	4.13	14.40
2016	8.56	1.00	1.43	0.56	0.70	0.15	0.34	0.28	1.78	6.46	17.83	13.02
2017	5.16	3.83	2.53	0.64	0.10	0.23	0.60	0.85	3.30	5.24	17.25	10.73
2018	4.58	4.25	3.65	0.64	0.50	0.40	0.72	1.05	1.55	4.52	13.95	11.20
**IFV**												
2015	22.80	48.85	45.28	25.96	3.10	0.33	0.14	0.00	0.38	0.10	0.48	1.53
2016	15.24	47.55	36.95	26.60	5.00	0.70	0.22	0.28	0.25	0.40	0.80	35.50
2017	26.20	7.70	8.95	12.72	5.03	2.20	1.10	0.55	0.93	0.66	4.05	41.55
2018	58.50	35.63	10.55	3.46	2.80	1.08	0.50	0.30	0.58	1.78	11.15	35.35
**HCoV**												
2015	8.74	1.40	0.95	0.56	0.10	0.50	0.58	0.60	0.38	1.38	1.80	5.53
2016	7.96	3.98	4.95	4.26	4.90	3.08	3.94	4.53	4.68	4.24	7.80	9.82
2017	10.26	6.45	5.15	3.62	2.68	2.35	1.02	1.45	1.55	2.14	5.15	7.20
2018	6.76	9.88	7.73	3.52	0.85	0.48	1.00	3.13	2.60	5.80	10.93	9.83
**HRV**												
2015	8.80	7.73	10.08	15.84	17.10	19.30	21.62	18.10	25.40	28.82	29.03	16.78
2016	8.52	3.78	12.28	15.44	19.55	16.38	19.62	19.60	18.40	24.56	20.68	8.02
2017	9.56	15.40	20.90	18.66	14.78	17.78	21.40	30.63	33.10	24.68	23.88	6.83
2018	2.52	5.95	20.63	28.34	21.53	20.18	21.14	12.70	24.63	22.08	15.70	9.78
**HBoV**												
2015	0.58	0.70	0.68	5.18	9.05	8.18	2.46	0.18	0.00	0.56	0.23	0.55
2016	1.06	0.83	1.93	3.86	4.55	2.80	1.06	1.15	0.83	0.30	0.25	0.30
2017	0.58	1.00	1.35	4.66	9.33	3.98	0.82	0.30	0.25	0.18	0.20	0.58
2018	0.26	0.70	0.10	0.88	4.35	7.18	5.78	0.55	0.63	0.60	0.85	0.88
**HMPV**												
2015	0.66	0.40	0.98	3.64	7.10	4.20	1.48	0.33	0.13	0.46	0.43	1.68
2016	3.56	4.20	8.05	12.64	8.53	2.83	0.66	1.20	0.98	0.42	1.05	1.14
2017	4.76	11.10	16.38	14.14	4.80	0.63	0.56	0.10	0.00	0.76	0.60	0.28
2018	0.86	2.95	8.60	19.12	15.33	6.00	2.82	0.73	0.48	0.08	0.18	0.15
**Group A Rotavirus**												
2015	14.10	22.35	28.90	16.38	6.33	2.70	1.72	3.25	5.38	2.62	1.65	0.98
2016	5.66	16.45	21.08	13.28	6.03	3.73	2.30	4.20	3.43	1.86	1.68	2.76
2017	10.38	21.68	30.33	20.26	8.13	5.53	1.68	2.30	3.45	2.34	4.35	7.38
2018	14.52	19.53	16.88	10.10	4.38	4.55	1.84	1.98	0.85	1.90	3.13	4.10
**Norovirus**												
2015	29.20	15.30	13.35	5.82	7.53	6.73	4.86	3.20	6.18	10.36	26.28	43.98
2016	38.62	21.85	20.33	13.64	7.80	4.08	3.66	3.40	2.65	9.32	28.58	41.06

2017	30.28	20.00	21.08	19.18	18.58	10.40	3.72	6.20	4.23	8.06	33.40	39.58
2018	25.46	19.20	10.78	15.74	9.30	9.90	4.34	3.20	5.35	6.28	14.95	16.90
**Enteric Adenovirus**												
2015	1.68	3.03	1.55	1.70	2.30	1.05	0.28	0.00	0.60	2.22	0.55	1.80
2016	2.28	2.58	1.25	2.30	2.28	2.75	2.16	3.43	10.73	6.88	5.68	6.78
2017	2.10	2.23	0.55	2.86	2.90	3.05	2.78	3.30	3.50	5.04	3.43	1.13
2018	2.40	1.85	2.55	3.04	2.85	5.65	4.44	6.45	6.03	3.82	3.33	2.15
**Astrovirus**												
2015	2.30	1.28	2.30	2.68	3.25	3.05	0.36	0.58	1.73	1.14	0.70	0.68
2016	0.94	1.18	2.48	3.20	3.40	4.30	2.52	2.28	3.43	2.82	2.05	0.86
2017	11.96	1.15	1.73	5.54	3.90	4.08	3.32	2.30	1.78	2.08	0.68	2.58
2018	0.94	1.73	0.00	1.82	3.05	2.50	2.74	5.18	5.63	3.98	2.73	2.33
